# Negative stereotypes of the Scottish diet: A qualitative analysis of deep-fried Mars bar references in bestselling newspapers in Scotland, 2011–14

**DOI:** 10.1016/j.appet.2016.04.030

**Published:** 2016-08-01

**Authors:** Christine Knight

**Affiliations:** Science, Technology & Innovation Studies, University of Edinburgh, Old Surgeons' Hall, High School Yards, Edinburgh EH1 1LZ, Scotland, UK

**Keywords:** Scotland, UK, Media, Discourse, Qualitative, Stereotypes, Culture, Scottish effect, Glasgow effect

## Abstract

The Scottish diet is associated in the UK media and popular discourse with unhealthy deep-fried foods. In addition to the stereotype's negative effects on perceptions of Scottish food, culture and people, there is evidence that the stereotype of the Scottish diet has negative effects on food behaviour and public health in Scotland, having been shown to encourage consumption of deep-fried foods and discourage positive dietary change. The most notorious deep-fried food associated with Scotland is the deep-fried Mars bar (DFMB), arguably invented in Stonehaven (near Aberdeen), and first reported in the Scottish and UK press in 1995. This article reports findings from an analysis of newspaper references to the DFMB in the two highest selling newspapers in Scotland, the Scottish Sun and the Daily Record, between 2011 and 2014. A keyword search (“deep fried Mars bar”) using the online media database Lexis Library generated 97 unique records, and the resulting dataset was analysed thematically and discursively. Analysis showed that both newspapers clearly associated the DFMB with Scotland. Further, both newspapers portrayed the DFMB and the broader “deep-fried” Scottish diet stereotype ambivalently (mixed positive and negative associations). However, the Daily Record actively criticised the DFMB stereotype much more often than did the Scottish Sun. These findings suggest that the Scottish population encounters different messages in the press about food and nutrition from people elsewhere in the UK, and that these messages vary depending on choice of media in Scotland. Given the known negative effects of the stereotype, differences in Scottish media discourse should be considered a potential factor in persistent health inequalities affecting Scotland. Educational efforts, and opening discussion with journalists and amongst the Scottish public, may be helpful.

## 1 Introduction

The Scottish diet has long been associated in the UK media, public discourse, and popular perceptions with unhealthy deep-fried foods. These include iconic items such as the deep-fried Mars bar, deep-fried pizza, and deep-fried haggis, as well as deep-fried pies and burgers, other deep-fried chocolate bars, and fish and chips. The most notorious of these is undoubtedly the deep-fried Mars bar (DFMB), which the Carron Fish Bar (formerly the Haven) in Stonehaven claims to have invented in the 1990s (Dow, 1995). In 2004, a survey of Scottish fish and chip shops found that 22% sold DFMBs, and 17% had done so in the past. Researchers concluded that “Scotland's deep-fried Mars bar is not just an urban myth” (Morrison & Petticrew, 2004: 2180).

The DFMB was first reported in the UK press in 1995 (Dow, 1995), generating significant national publicity (eg, Arlidge, 1995, Low, 1995), and instigating an ongoing series of references to the DFMB in the UK media that has lasted 20 years. Such publicity, which also includes the promotion of the DFMB to international tourists to Scotland as a local specialty (eg, Rough Guides, 2016), has no doubt increased availability and demand. Nonetheless, outside the UK there is very limited association of the DFMB or other deep-fried foods specifically with Scotland. The DFMB and other deep-fried chocolate bars are certainly sold in other countries around the world, including widely at state fairs in the United States (Smith, 2013: 306; see also Hirsh, 2010, Fritsch, 2012, Gamble, 2012).

In incidental findings from previous interview studies with people in Scotland, the stereotype associating the Scottish diet with deep-fried foods has been shown to have negative effects on food behaviour. This includes discouraging positive dietary change (Fuller, Backett-Milburn, & Hopton, 2003: 1045S), and encouraging consumption of deep-fried foods by young people from minority ethnic groups in order to “claim” Scottish national identity (Hopkins, 2004: 265). These impacts on health behaviour are in addition to the stereotype's negative effects on perceptions of Scottish food, culture, and people (by Scots themselves and others).

Stereotyping of the Scottish diet by English writers and cartoonists dates back to at least the eighteenth century, and has often surfaced at times of particular political tension between the two nations (Fraser, 2011). However, such stereotypes have taken on a new form and new significance in the contemporary context of concerns about obesity and nutrition-related disease, notably heart disease (Knight, 2016). The relationship between these real problems of public health in Scotland (discussed further below) and stereotypical patterns of representation is the focus of this paper, which constitutes the first investigation of this topic. It asks: What messages do people in Scotland receive via the media in relation to the “deep-fried” stereotype of the Scottish diet?

Scotland has some of the highest rates of obesity, and diet-related disease such as heart disease, worldwide. Most recent available figures for OECD nations place Scotland 5th in the world for overweight and obesity combined, and 6th for obesity alone (Castle, 2015: 19–20). Moreover, Scotland faces serious health inequalities, with marked disparities in mortality and life expectancy in the West of Scotland (in particular) relative to other parts of the UK and comparable developed nations. Although much of this excess mortality can be accounted for by socio-economic deprivation, a significant proportion remains unexplained – a phenomenon known as the “Scottish effect” or “Glasgow effect” (Landy et al., 2012, McCartney et al., 2011, Popham and Boyle, 2011, Walsh et al., 2010a, Walsh et al., 2010b). These problems of Scottish public health, and the widespread attention they have received in the UK media, are both relevant to this study. They are also significant policy concerns for the Scottish Government, with corresponding investment in addressing them (Scottish Government, 2010).

Previous qualitative research on diet and nutrition in Scotland has often been situated within the wider British public health and social context (eg, Backett-Milburn et al., 2006, Backett-Milburn et al., 2010, Jones et al., 2007, Wills et al., 2006), rather than a specifically Scottish context. Thus there has generally been a lack of attention to national or regional identity, culture or place in examining nutrition and public health discourse(s) in Scotland. A notable exception is the work of O’Brien, Hunt, and Hart (2009) exploring how men in the West of Scotland (around Glasgow) constructed their health beliefs and behaviours in the focus group setting, in relation to masculinity. The quotation used in the title of their paper, “The average Scottish man has a cigarette hanging out of his mouth, lying there with a portion of chips”, clearly indicates the centrality of deep-fried foods to ambivalent stereotypes of unhealthy masculinity in (the West of) Scotland, and ties their consumption to other unhealthy practices such as smoking and a sedentary lifestyle. Many participants distanced themselves from this version of West of Scotland masculinity: “Participants expressed a mix of horror, disgust and amusement in discussions of the excesses of the ‘junk food diet’ (particularly the deep-fried Mars Bar) and other poor lifestyle ‘choices’ they had observed in Glasgow” (371). However, others appeared to recognise that the version of masculinity that such behaviours represent remained “desirable” and “exalted” in working-class Glasgow (371).

Likewise, Emslie and Hunt (2008) report that participants in the West of Scotland related men's health behaviours to broader damaging stereotypes of unhealthy “macho” masculinity in the region, involving poor diet, drinking, smoking, and low physical activity (812-15). Relatedly, Haddow, Mittra, Snowden, Barlow, and Wield (2014) identify the national stereotype of Scotland as the “sick man of Europe” (5–6), and argue that a second narrative has emerged more recently in which Scotland is marketed as a “living lab” (9–10): an ideal place to conduct medical research, especially genetic and genomic research, because of its sick population and low rates of migration. Finally, a study in the Western Isles highlights another specific regional discourse within Scotland in relation to diet and nutrition: McKie, Clark, MacLellan and Skerratt (1998) found that participants reported a “traditional island diet” (pre-World War 2) that included significant quantities of fried and high-fat foods (377). Participants suggested, however, that this diet was wholesome and healthy overall because these fried/high-fat components were balanced by high levels of physical activity (377).

Research on the representation of diet and nutrition in the UK media aligns with work carried out elsewhere. In particular, nutrition information in the press is generally inaccurate (the problem being more pronounced in “tabloid” than in “broadsheet” newspapers) (Cooper, Lee, Goldacre, & Sanders, 2012), and there was increasing coverage of obesity in the 1990s and 2000s, with studies showing a peak in coverage variously between 2006 and 2008 (Hilton et al., 2012, Ries et al., 2011). Importantly for this study, different media in the UK report obesity and public health differently. For example, “quality” newspapers (historically, those in broadsheet format) are more likely to highlight societal factors as causes of obesity (Hilton et al. 2012: 1694), and an earlier study on the reporting of public health policy found that quality newspapers in Scotland (the Scotsman and Glasgow Herald) were more likely to highlight health inequalities (Davidson, Hunt, & Kitzinger, 2003).

Useful insight into the significance of class and region in UK media discourses about diet can be gleaned from research on the reality television series *Jamie's Ministry of Food* (JMF), in which celebrity chef Jamie Oliver attempts to improve the culinary skills and diet of working-class people in the Yorkshire town of Rotherham. Hollows and Jones (2010) locate JMF “within a wider discourse of class pathologization” in the UK (308), and Warin (2011) further links this to the programme's construction of place, pointing out that “Oliver taps into stereotypical discourses of life in a northern English town”: his “approach feeds directly into a well-established discourse in the United Kingdom about the north-south divide, in which northerners are represented as in a constant state of post-industrial degeneration and stuck in impoverishment” (29).

Finally, scholars in film, cultural and media studies highlight the tendency to represent Scotland (including by Scottish writers and producers themselves) through a negatively stereotyped representation of Glasgow as a place of “multiple deprivation and crime”, which can be traced back to nineteenth-century industrialisation (Blain & Burnett, 2008: 8–9). On other topics (such as sport), research on the representation of Scotland in the UK media shows that tensions between England and Scotland are very much “live” in newspaper reporting (Bloyce, Liston, Platts, & Smith, 2010), and London-based UK media outlets are prone to derogatory, stereotypical representations of Scotland and Scots (Reid, 2010). The Scottish media “push back” against these representations (Reid, 2010), and (beyond the context of sports reporting) Scottish editions of the London-based newspapers “alter stereotypes or negative judgements to suit the imagined national audience” (Rosie, MacInnes, Petersoo, Condor, & Kennedy, 2004: 451). For example, Rosie et al. (2004) discuss a column in the Daily Mail by Ephraim Hardcastle (24 October 2000) which compared a Scottish MP's “thick” accent to porridge. They show that the version in the Scottish Daily Mail contained small but significant changes: “Whereas in the version sold in England, the point of the story was to deride the speaker's accent, in the version sold in Scotland, the point of the story could be read as a derisory comment on English prejudice” (Rosie et al. 2004: 452).

## 2 Methods

Two newspapers were used for this study: the Daily Record and the Scottish Sun (including their respective Sunday titles, the Sunday Mail and Scottish Sun on Sunday). Selection was based on the Audit Bureau of Circulations' newspaper rankings for Scotland (All Media Scotland, 2014), which show that these two newspapers enjoy by far the highest sales of any in Scotland, well ahead of their nearest competitor the Scottish Daily Mail. (The Sunday Post competes with these two newspapers in Sunday sales, but has no daily equivalent.) This study used newspaper archives (rather than radio or television) because they are readily accessible and searchable; radio and television archives for Scotland are limited, costly to access, and not always indexed to the level of detail required for this study.

Newspapers sold in Scotland fall into two categories: “indigenous” Scottish newspapers (produced in Scotland and circulated almost exclusively in Scotland) and Scottish editions of the major UK national newspapers (the “Fleet Street” dailies). In the latter category, Scottish newspaper editions vary in terms of how much content is changed for a Scottish audience (Rosie et al. 2004: 440). The Daily Record is an “indigenous” Scottish newspaper, while its rival the Scottish Sun is the (heavily Scotticised) Scottish edition of English newspaper the Sun. Both newspapers are tabloids. The Daily Record supports Labour. The Sun, despite dalliances with the Conservatives and (in Scotland) the Scottish National Party (Hutchison, 2008: 62), supported Labour during the period covered by this study; it would be more accurately described as populist than left-wing. Established in 1895, the Daily Record is known for presenting a specifically Scottish perspective and is especially popular in Glasgow (Law, 2001: 305–306). Despite being a more recent “interloper” (Law, 2001), the Sun has invested significantly in its Scottish edition, resulting in great success in the Scottish market (Rosie, Petersoo, MacInnes, Condor, & Kennedy, 2006: 330–331; Hutchison, 2008: 67).

The study used Lexis Library, the main online database for UK newspaper articles. Access to the Daily Record is straightforward. However, Lexis Library has not always distinguished between different national editions of the Sun: it is only possible to identify articles from the Scottish Sun consistently from 2011. 1 January 2011 was therefore set as the start-date for the study to enable comparison between the Scottish Sun and Daily Record. An end-date of 31 December 2014 was set in order to capture representation patterns around the Scottish Independence Referendum in September 2014, as well as coverage of the Glasgow Commonwealth Games in July–August the same year. The four-year period was sufficient to capture an appropriate dataset for qualitative analysis.

This study focussed specifically on the deep-fried Mars bar as this is widely represented as the epitome of the unhealthy Scottish diet, and preliminary searches identified many more references to the deep-fried Mars bar than other stereotypically Scottish deep-fried foods, such as deep-fried pizza or haggis. (In the Daily Record/Sunday Mail, there were 3 articles that referred to deep-fried pizza and 1 that referred to deep-fried haggis during the study period. In the Scottish Sun/Sun on Sunday, the figures were 7 for deep-fried pizza and 0 for deep-fried haggis.) I therefore searched Lexis Library using the search term “deep fried Mars bar” for the two selected newspapers. The search returned 54 articles from the Daily Record/Sunday Mail, and 43 articles from the Scottish Sun/Sun on Sunday, resulting in a full dataset of 97 articles.

I undertook thematic analysis assisted by NVivo 10 qualitative analysis software. As the first investigation of this topic, and therefore to some extent exploratory, I coded mainly according to emerging themes. However based on my research question, I paid specific attention to, and coded for, positive/negative representations of the DFMB, as well as associations with place (Scotland or elsewhere). Whilst the findings reported here are based primarily on thematic analysis, I simultaneously undertook discourse analysis (annotating this within NVivo) – using the term here in the wider sense employed in cultural and media studies. This denotes analytic methodologies concerned with the way in which social phenomena are constructed through language (Silverman, 2006: 223-24), and involves close attention to how particular words and phrases produce meaning. The combination of analytic methods allowed me not just to identify key media messages but also to explore layers of implicit meaning.

## 3 Results

Both the Daily Record and the Scottish Sun used the deep-fried Mars bar humorously and ironically, while clearly associating it with Scotland. Both newspapers portrayed the deep-fried Mars bar and the “deep-fried” Scottish diet stereotype ambivalently, with mixed positive and negative associations. However, the Daily Record actively criticised the deep-fried Mars bar stereotype much more often than did the Scottish Sun.

### 3.1 Humour and irony

Humour and/or irony are almost universal in references to the deep-fried Mars bar, regardless of whether these are otherwise positive or negative. In many cases humour and/or irony is introduced by the journalist. For example, in an article on unusual sandwich fillings, marking National Sandwich Week, the deep-fried Mars bar is used as an ironic benchmark for the limit of good (or bad) taste:

Scots are a sandwich short of a picnic when it comes to packing our pieces [sandwiches] with foul fillings. Never mind the deep-fried Mars bar – step forward the BEANS AND BANANA toastie! (18 May 2012, Scottish Sun)

Similarly, an op-ed about the deep-fried Mars bar opens with an overt joke, linking Scotland's notoriously poor national football performance to the nation's equally notorious predilection for deep-fried foods:

An English mate always jokes that the Scotland football team never makes it to the World Cup Finals because the players eat too many deep-fried Mars bars. What utter garbage. Everybody knows it's too many deep-fried pizzas that stifle our qualification. (7 September 2012, Scottish Sun)

In other cases, rather than being introduced by the journalist, the humour and/or irony associated with the deep-fried Mars bar is intrinsic to the event or news that is being reported. For example, the Daily Record reports that a London restaurant is using the deep-fried Mars bar in a form of gastronomic irony – resulting from the contrast between the poor nutrition, taste and class connotations of the deep-fried Mars bar and the upmarket London restaurant context and price-tag:

The deep-fried Mars bar, the edible byword for the notoriously unhealthy Scottish diet, is on the menu of a top London restaurant. The Duck and Waffle, on the 40th floor of one of London's new skyscrapers, makes the *ironic offer* for the tidy price of £7 a portion. (1 April 2013, Daily Record, emphasis added)

Although the date of this article may suggest an additional layer of humour at work, the Duck and Waffle is a real (and highly regarded) London restaurant, and numerous reviewers and food bloggers testify to the quality of its deep-fried Mars bar (eg, Delane, 2013, Shaw, 2013).

### 3.2 Association with Scotland

As all three examples above illustrate, both the Daily Record and the Scottish Sun clearly associate the deep-fried Mars bar with Scotland. This is achieved through a range of mechanisms. Most simply, analysis identified numerous adjectival and possessive grammatical constructions of the deep-fried Mars bar as Scottish or belonging to Scotland. For example, various journalists across diverse articles described the deep-fried Mars bar as a “Scottish delicacy” (eg, 6 September 2012, 10 November 2014, Daily Record), “Scots delicacy” (eg, 30 July 2012, Scottish Sun) or “Scotland's most infamous fast-food snack” (12 June 2012, Daily Record).

Other discursive mechanisms linking the DFMB to Scotland were more complex. For example, articles reported visitors to Scotland being encouraged to try the deep-fried Mars bar as a quintessentially Scottish dish – as here in a reported interchange between British Prince William and Australian swimmer Alicia Coutts on the prince's Australian tour:

The prince was introduced to Australian swimmer Alicia Coutts, who will be competing in the Glasgow Commonwealth Games. She explained: ‘I said that I'd been told I should sample Irn-Bru. But the prince said that it's overrated and that I should try deep-fried Mars bars.’ (17 April 2014, Daily Record)

Analysis also identified several examples of Scottish expatriates reportedly feeling nostalgia for the deep-fried Mars bar, along with other foods and drinks associated with Scottish national identity (such as haggis, or the hugely popular and iconic Scottish soft drink Irn-Bru):

Doctor Who actress Karen Gillan says she misses haggis and deep-fried Mars bars after moving to the US. Inverness-born Karen […] has been filming in Alabama for a thriller called Oculus. She said: […] ‘I loved Alabama because it reminds me of my home town. There is no one there and there is a lot of fried food. My guilty pleasure is Mars bars put in the deep fat fryer.’ (4 November 2012, Sunday Mail)

Likewise, at home in Scotland, reporters and interviewees explicitly identified the deep-fried Mars bar as a typical Scottish food and drink product, akin to whisky in its national (and even nationalist) associations. The following excerpt quotes Scot James Morton, a finalist on BBC television cooking competition the Great British Bake Off:

James is proud to fly the flag for Scottish baking […]. He said: ‘I am delighted to have done my bit for Scotland. I tried to use as many Scottish ingredients as possible but had to lay off after I got a telling-off about the strength of the whisky I used in my jelly one week. My only regret was not incorporating a deep-fried Mars bar.’ (14 October 2012, Daily Record)

The national association of the deep-fried Mars bar with Scotland occurred almost three times as often as specific references to the town of Stonehaven, as the “hometown of the deep-fried Mars bar” or “the birthplace of the deep fried Mars bar”. There were also a small number of references in both newspapers to the deep-fried Mars bar in association with Glasgow and the West of Scotland. For example, an interviewee quoted in an article about Glasgow during the Commonwealth Games said:

We're keen to show the world we're not just deep-fried Mars Bars and ‘see you Jimmy’ hats. We're a stylish, sexy and fun city. (2 August 2014, Scottish Sun)

In a second example, this association appears in an article about Spanish football team La Hoya Lorca, which uses images of its local food product, broccoli, on its uniform. The reporter ironically suggests similar options for Scottish football teams, including deep-fried Mars bars for the Glasgow football club Partick:

The Segunda B outfit [La Hoya Lorca] have unveiled a barf-tastic change strip using a photoprint of some broccoli heads. That's right, a large picture of real broccoli with arms on and a number on the back. It's meant to reflect the veg-growing prowess in its province of Murcia. […] This is probably the first non-abstract kit in history, let alone the first foodie one – and could catch on here in Scotland, using nature's bounty. Caley in thistles? Peterhead in fish scales? Ayr in soil-kissed potatoes? St Johnstone in raspberries? Forfar in bridies? Arbroath in smokies? Partick in deep-fried Mars Bars? (14 October 2013, Scottish Sun)

### 3.3 Positive portrayals

In both the Daily Record and the Scottish Sun there are numerous positive portrayals of the deep-fried Mars bar, for example in relation to its sensory pleasures and (perhaps unexpected) nutritional benefits. The following examples are from well-known celebrity chefs in the UK, Englishwoman Nigella Lawson, and Italian Gino D'Acampo, who moved to England in 1995:

Nigella Lawson has revealed her recipe for looking young – doughnuts and deep-fried bars of chocolate. The curvy TV chef, 52, said: ‘I'm a great believer in fat. My view is that you moisturise from the inside. I like any doughnut that's going. I like anything in batter or that is deep fried. I have not only had a deep-fried Mars Bar, I have also done a recipe for deep-fried Bounty. Very delicious, typically tropical.’ (12 October 2012, Daily Record)

Gino [D'Acampo] is an excited advocate for Scottish food – salmon, seafood and meat are all part of his diet. And he's even tried a deep-fried Mars bar. He shrugged: ‘If you've been drinking too much, it definitely helps. I had one in Edinburgh six or seven months ago when I was filming. I'd had three or four pints at the time and it was really good. It was cold outside and the deep-fried Mars bar was warm, and there were plenty of calories.’ (22 June 2013, Daily Record)

In these examples, dietary fat, calories, and warmth are represented as pleasurable and valuable food qualities, especially (in the second excerpt) in the Scottish climate. Whilst Nigella is arguably promoting her own recipe for deep-fried Bounty, both these England-based chefs may be seen to be currying favour with Scottish audiences. The significance of these excerpts lies in the fact that the deep-fried Mars bar is a potential vehicle for doing so, and is treated as such not just by Lawson and D'Acampo, but also by the Daily Record.

Endorsements of the deep-fried Mars bar by celebrities from England or abroad are common in both newspapers. For example, English singer Jessie J is quoted in an interview: “I always have so much fun in Scotland. The crowds are always so amazing and I love a deep-fried Mars bar!” (1 July 2014, Daily Record). Likewise, English rapper Dizzee Rascal is reported as saying:

I used to go to this chip shop with a Mars and 20p, and they'd batter the Mars. It was like the best dessert. It sounds disgusting but the way it melts in your mouth is incredible. (12 June 2012, Daily Record)

This latter example again highlights the sensory, textural pleasures of the deep-fried Mars bar.

### 3.4 National pride

Positive portrayals and endorsements of the DFMB in the Scottish press imply a sense of Scottish national pride in the association of the DFMB with Scotland – that is, they implicitly suggest that the Scottish nation can or should be proud of the DFMB. National pride is also implied in other excerpts discussed above, such as the reporting of quotations from James Morton and Alicia Coutts. Analysis of data from both the Daily Record and the Scottish Sun also identified examples of Scots directly expressing pride in the DFMB as a Scottish dish or invention – sometimes, but not always, ironically. For example, the Carron Fish Bar's Lorraine Watson is quoted in overt patriotic defence of the DFMB against an English patent threat:

A Londoner is trying to slap a patent on Scotland's other national dish – the deep-fried Mars Bar. Camden firm Crispy Candy claim they've come up with a better version. […] But Lorraine Watson, of Stonehaven's Carron Fish Bar, said: ‘No matter what happens, the deep-fried Mars Bar was born here and is and always will be Scottish. The way we do it works, and will continue to be popular with everyone that visits for years to come.’ (20 September 2013, Daily Record)

The reference to the deep-fried Mars bar as “Scotland's other national dish” implicitly couples the DFMB with hugely popular and iconic Scottish soft drink Irn-Bru, known as “Scotland's other national drink” – discursively elevating the DFMB to the same level. The implied ironic comparison is with Scotland's established national food and drink pairing, haggis and whisky.

In a further example of overt national pride, in this case clearly ironic, the Scottish Sun quotes character Bruce Robertson from Scottish novel Filth (Welsh, 1998), recently made into a film:

In the immortal words of Filth's Bruce Robertson, ‘We're the country who brought the world television, the steam engine, golf, whisky, penicillin and of course, the deep-fried Mars bar.’ (12 October 2013, Scottish Sun)

This excerpt is from an article about the achievements of Scots in the film industry, and ironically elevates the DFMB to the status of other Scottish scientific and cultural inventions and achievements.

### 3.5 Negative portrayals

Set against these positive and proud portrayals however, there is roughly equivalent content in both the Daily Record and the Scottish Sun presenting the DFMB as emblematically unhealthy or grotesque. For example, analysis identified numerous passing references to the DFMB as “the artery-clogging Scottish delicacy” (10 November 2013, Daily Record), “calorie-busting snack” (7 September 2012, 18 September 2012, Scottish Sun), or similar. More extensive examples of negative portrayal included this report on a clinical trial on the effects of deep-fried Mars bars:

Eating a deep-fried Mars bar can raise the risk of having a stroke within minutes, doctors claim. Scotland's unhealthiest snack – a whopping 1200 calories – is so full of fat that it slows the supply of blood to the brain. (28 September 2014, Daily Record)

A final example, quoting English television presenters Ant and Dec, illustrates how negative press coverage links nutrition and taste, intertwining references to sensory and physical disgust and perceived health risks (here, cardiovascular disease):

Ant & Dec love Scotland. Just don't ask them to tuck into our controversial delicacy, the deep-fried Mars Bar. Gagging at the memory, Dec said: ‘Revolting. It's a heart attack waiting to happen. It's really not good.’ Jumping in, Ant added: ‘We tried one. It's rank.’ (24 March 2012, Daily Record)

### 3.6 Critique

As well as these general negative representations, analysis identified a strong strand of critique of the DFMB stereotype, especially in the Daily Record. This critique took three forms. First, some articles (or commentators quoted within them) simply contradicted the deep-fried Mars bar stereotype, denying its veracity. Here, for example, Member of the Scottish Parliament Jim Eadie responds to claims about the Scottish diet by an English contestant on reality television show The Apprentice:

Adam Corbally, 32 […] says: ‘Scots generally eat deep fried Mars Bars and deep fried food.’ Last night Edinburgh Southern MSP Jim Eadie said: ‘We get a wee bit tired of these kind of comments. I'm sure the good people of Edinburgh will be only too happy to show them that the diet does not conform to the stereotype […]’ (23 April 2012, Daily Record)

A second strategy observed was to name and identify the DFMB as stereotype, thus implicitly critiquing its authenticity. In this mode, the DFMB is often critiqued alongside other (usually negative) stereotypes of Scottish culture, as in the following two excerpts:

Here we take a look at some of the most cringeworthy examples of Scots stereotypes on film, telly and radio: JIMMY WILSON The star of video game The Darkness II is a permanently drunk thug with ginger hair. He belches a lot and hates the English. […] All that's missing from Jimmy's armoury of stereotypes is a deep-fried Mars bar in a holster. (29 December 2011, Daily Record)

Godwin's Law states the longer an online conversation grows, the greater the chances of someone mentioning Hitler becomes. No one has named the algorithm stating the longer a conversation about Scotland grows in London, the more chance of a mention for Braveheart, deep-fried Mars Bars, The Proclaimers and ‘subsidy junkies’. (13 December 2014, Daily Record)

Within this second mode, the writer might also explicitly contest the DFMB stereotype, as in the following example by well-known Scottish radio and television presenter Shereen Nanjiani, writing in the Scottish Sun:

Leaving aside the tiny issue of whether Mars bars can ever be considered healthy, I'm more bothered about what the artery clogging deep-fried version has done to our reputation as one of the unhealthiest nations in the world. I'm fed up telling people from down south, and recently from Canada, that deep-fried Mars bars are NOT our national dish. It's been a success story for the Carron [Fish Bar], and good luck to it, but let's not endorse another negative stereotype of our country. (10 September 2012, Scottish Sun)

The third and most frequent critical strategy identified was to highlight Scottish gastronomic and culinary culture (high-quality Scottish produce, restaurants and chefs), in explicit contrast to the DFMB stereotype. For example, English Masterchef presenter Gregg Wallace is quoted rhapsodising about Scottish produce (meat, fish and berries) and traditional Scottish dessert cranachan (made from raspberries, cream, oats, honey, and whisky):

From a greengrocer's perspective, Scotland has me in raptures. They grow the best soft fruit in the world and have ample supplies of the most exquisite lamb, beef, fish and game. It's only uneducated folk that associate the country with deep-fried Mars bars – which I have tried. Give me Cranachan any day of the week. (18 May 2011, Daily Record)

The following excerpt describes BBC television show the Incredible Spice Men, featuring Edinburgh chef Tony Singh, again explicitly contrasting his culinary flair with the DFMB stereotype:

The spice boys slipped a couple of handfuls of whole aniseeds into a pan of bonfire toffee, which went down a storm, added orange and cardamom to bread and butter pudding and sprinkled cinnamon and chilli on vanilla ice cream, with thumbs up all round. Definitely one in the eye for anyone who still believes Scottish haute cuisine is a deep-fried Mars bar. (25 August 2013, Daily Record)

Likewise, the introduction to an article about Scottish Food and Drink Fortnight explicitly contests the DFMB stereotype with reference to high-quality Scottish produce, producers, and Scottish celebrity chef Nick Nairn:

Scots' reputation as lovers of deep-fried Mars Bars, Irn-Bru and greasy fish suppers [fish and chips] is history. Our fruit, vegetables and even chocolate are considered among the world's finest and quality produce is now big business. Scottish Food and Drink Fortnight runs until September 22 to promote our produce. Amanda Keenan meets three food heroes whose home-grown fare is in demand worldwide, while our Saturday magazine chef Nick Nairn […] shows how to make the best of our produce with three of his favourite recipes. (10 September 2013, Daily Record)

## 4 Discussion

This study found that the Daily Record and the Scottish Sun, the two highest selling newspapers in Scotland, clearly associated the deep-fried Mars bar with Scotland. The two papers portrayed the DFMB and broader “deep-fried” Scottish diet stereotype ambivalently. The Daily Record criticised the deep-fried Mars bar stereotype much more often than the Scottish Sun. In the context of a well-established and pervasive negative stereotype of the Scottish diet across the wider UK media, it thus appears that these newspapers for the Scottish market have followed two contradictory possible lines of response: on the one hand, to embrace the DFMB stereotype, portraying it as positive and a source of national pride; on the other, to disown the DFMB and contest the stereotype. As indicated, the latter path was followed somewhat more by the “indigenous” Scottish paper the Daily Record.

These results have three important implications. First, people in Scotland are receiving specific nutrition and health messages in the media, with the two highest selling newspapers for the Scottish market associating deep-fried foods (notably the DFMB) particularly with Scotland. Second, the messages that newspaper readers in Scotland encounter in relation to the “deep-fried” stereotype of the Scottish diet are mixed (positive and negative) and potentially confusing. Third, choice of media in Scotland makes a difference to the messages readers encounter about the Scottish diet: different newspapers contest the negative Scottish diet stereotype to different extents. These findings thus highlight the specificity of media discourses about nutrition and diet in Scotland, and the need to attend to the distinct Scottish media, and the particularities of diet discourse in Scotland, in further research – whether about diet and health in the UK media, or diet and health discourse in Scotland.

These findings to some degree support those scholars who highlight the “sick man of Europe” stereotype of Scotland (Haddow et al. 2014), while also supporting previous research showing that Scottish newspapers contest derogatory stereotypes of Scotland (for example, in relation to sport) (eg, Reid, 2010). Drawing these two strands together, these findings point to complex patterns in which Scottish national pride is implicated in divergent responses to negative health stereotyping of Scotland. In this data national pride was clearly mobilised both in positive portrayals of the DFMB stereotype, but also the equally strong (if not stronger) drive to contest this stereotype and present healthier, classier images of Scotland and its food (especially in the Daily Record). My findings in relation to the Scottish press align with the ambivalence noted by O'Brien et al. (2009) in their Glaswegian focus groups' responses to the DFMB, which they describe as “a mix of horror, disgust and amusement” (371). These findings also tally with broader and longstanding assessments by scholars across disciplines of Scottish cultural ambivalence, although such a reading of Scottish culture has been questioned (McCrone, 2001: 127-48).

These results also offer further insight into regional nutrition discourses in Scotland. Alongside the association of the DFMB with Stonehaven in the North East, it appears that the DFMB has become attached to pre-existing stereotypes of multiple deprivation and poor health behaviours (especially amongst men) in Glasgow and the West of Scotland (Emslie and Hunt, 2008, O'Brien et al., 2009). This suggests that the DFMB stereotype has partially merged with longstanding negative cultural representations of Glasgow, and also illustrates the tendency for Scotland as a whole to be represented in terms of Glasgow (Blain & Burnett, 2008: 8–9).

In many cases, the Daily Record and Scottish Sun are clearly responding to wider British (and occasionally, international) use of the DFMB to stigmatise Scotland and its people by disparaging national food habits. This phenomenon incorporates moral, class, and taste judgements, as well as nutrition, although a full discussion is beyond the scope of this paper (see Knight, 2016 for analysis of the historical and cultural factors which enabled the DFMB slur to emerge and persist). The contribution of the present study is to show how the Scottish press respond to this in different ways, indicating that within stigmatised cultures or groups, stigma may simultaneously be resisted, contested, (re)appropriated, and embraced. This study therefore adds to the limited previous research internationally on nutritional stigma affecting minority or marginalised populations – for example, Everett's research (2009) on deep-fried foods and nutritional and moral stigma in Newfoundland and Labrador, Canada. Whilst the relationship between food and (national) identity is extremely complex and impossible to generalise (Scholliers, 2001), this research may thus have wider significance in illuminating similar patterns elsewhere.

The nationality of those quoted is important in understanding how the Scottish press represents the DFMB. Quotations from people well-known to be, or explicitly identified as, Scottish are relatively unproblematic. Quotations from English or international sources are somewhat more complex. Some celebrities (such as Nigella Lawson and Jessie J) may seek to “win over” Scottish audiences by expressing a liking for the DFMB, whilst others (such as Adam Corbally) may achieve the opposite by echoing the DFMB slur negatively or unwittingly. The fact that statements about the DFMB can be deployed as a mode of relation with Scottish audiences is in itself significant. For the purposes of this study, of further importance is the way in which these quotations are presented within the Scottish press, in bestowing approval or sanction on both the celebrity and the DFMB itself (as discussed in each case in the Results). Similar significance occasionally attaches to the nationality of the journalist or writer themselves – for example, Shereen Nanjiani writing as a well-known Scottish broadcaster. However, unless a print journalist has a particularly high profile, audiences are unlikely to know her or his nationality, and indeed journalists are not always named in the articles analysed here. In interpreting these findings, more important than the nationality of journalists themselves is the context in which they write and their imagined or intended readers, both the Daily Record and Scottish Sun being aimed specifically at a Scottish audience.

These findings may be specific to these two newspapers, given that both are tabloids marketed to a working-class Scottish audience. However, the fact that findings were generally similar across the “indigenous” Daily Record, and Scottish edition of the English-based Sun, suggests wider patterns. Moreover, the outstanding popularity and market share of these two newspapers in Scotland makes these findings significant in and of themselves. Results may have been different had the study extended back prior to 2011; the discourse of critique in the Daily Record in particular suggests growing Scottish cultural confidence in the lead-up to the independence referendum. However, while prior patterns of representation may well have had significant influence, the period covered here is the most recent and therefore most immediately influential on the Scottish public.

These findings suggest that distinct media messages about the Scottish diet may be a factor in unexplained excess morbidity and mortality in Scotland, and thus part of a vicious circle. Although difficult to demonstrate, this hypothesis warrants investigation. Thus future research might valuably consider whether choice of media amongst the Scottish population predicts health beliefs, behaviours or outcomes, independent of class and other known predictors (for example, through epidemiological surveys), as well as how people in Scotland respond to the deep-fried stereotype (for example, through qualitative interview or focus group research). Research across a wider range of media outlets (including newspapers, radio and television) may also be valuable, to validate findings and compare messages disseminated to different audiences – in particular, across different locations and socioeconomic groups.

## 5 Conclusion

This is the first study to trace diet and nutrition discourse in the Scottish media, providing valuable insight into distinct patterns of representation on this topic in Scotland. It thus adds to our understanding of nutrition discourses in the UK media and popular culture, bringing a specific Scottish perspective. It also adds a national Scottish frame to previous qualitative research studies on diet and health discourse in Scotland, which have tended to situate findings in the wider UK context, or (less often) to highlight regional identities and discourses.

These findings highlight the importance of sub-state national identities, relationships and tensions in UK health and nutrition discourses. These should not be ignored in considering and researching persistent, unexplained health inequalities within the UK, notably those affecting Scotland. Moreover, these findings have practical relevance for journalists and press offices, as well as for health promotion and education efforts in Scotland, to reduce the negative impact of the Scottish diet stereotype through awareness-raising and presenting alternative, more positive images.

Finally, although these findings are specific to Scotland, and Scotland's status as a sub-state nation within the UK, they have broader significance elsewhere in the world in understanding patterns of nutrition stigmatisation in the media and popular culture in relation to minority populations – whether defined by location, ethnicity, class, or other characteristics.
